# Shaping the genome of plants

**DOI:** 10.7554/eLife.54874

**Published:** 2020-02-06

**Authors:** Ajeet Chaudhary, Rachele Tofanelli, Kay Schneitz

**Affiliations:** 1 Plant Developmental Genetics, School of Life SciencesTechnical University of MunichFreisingGermany

**Keywords:** polyspermy, plant reproduction, sperm cell, egg cell, triploid block, *A. thaliana*

## Abstract

Fertilization of an egg cell by more than one sperm cell can produce viable progeny in a flowering plant.

**Related research article** Mao Y, Gabel A, Nakel T, Viehöver P, Baum T, Tekleyohans DG, Vo D, Grosse I, Groß-Hardt R. 2020. Selective egg cell polyspermy bypasses the triploid block. *eLife* **9**:e52976. doi: 10.7554/eLife.52976

Sexual reproduction involves an egg cell being fertilized by a sperm cell to form a new cell called a zygote. The egg cell and the sperm cell both contain one complete set of chromosomes, and thus one copy of the genome, so the zygote contains two sets of chromosomes and two copies of the genome. An egg cell can also be fertilized by multiple sperm – a process known as polyspermy – but this usually results in a zygote that is not viable.

Animals employ a number of different mechanisms to avoid eggs being fertilized by more than one sperm (Iwao, 2012). Plants also rely on a variety of mechanisms to avoid polyspermy (Grossniklaus, 2017; Scott et al., 2008; Tekleyohans et al., 2017), but the situation is more complicated because higher plants can undergo a unique process called double fertilization (Dresselhaus et al., 2016).

During double fertilization a single pollen tube carries two sperm cells to a structure called the ovule (Figure 1). As well as harbouring the egg cell, the ovule also contains a second female gamete called the central cell. In a coordinated mechanism one sperm cell fuses with the egg cell, and the second with the central cell (Sprunck, 2020). Once fertilized the egg cell grows into the embryo while the central cell develops into the endosperm, the tissue that nourishes the embryo (like the placenta in humans). In many species polyspermy is inhibited by suppressing the arrival of a second pollen tube at the ovule during successful fertilization. Occasionally, however, more than one pollen tube reaches the ovule via a phenomenon known as polytubey. This process can lead to polyspermy of the egg cell, the central cell or both.

**Figure 1.**
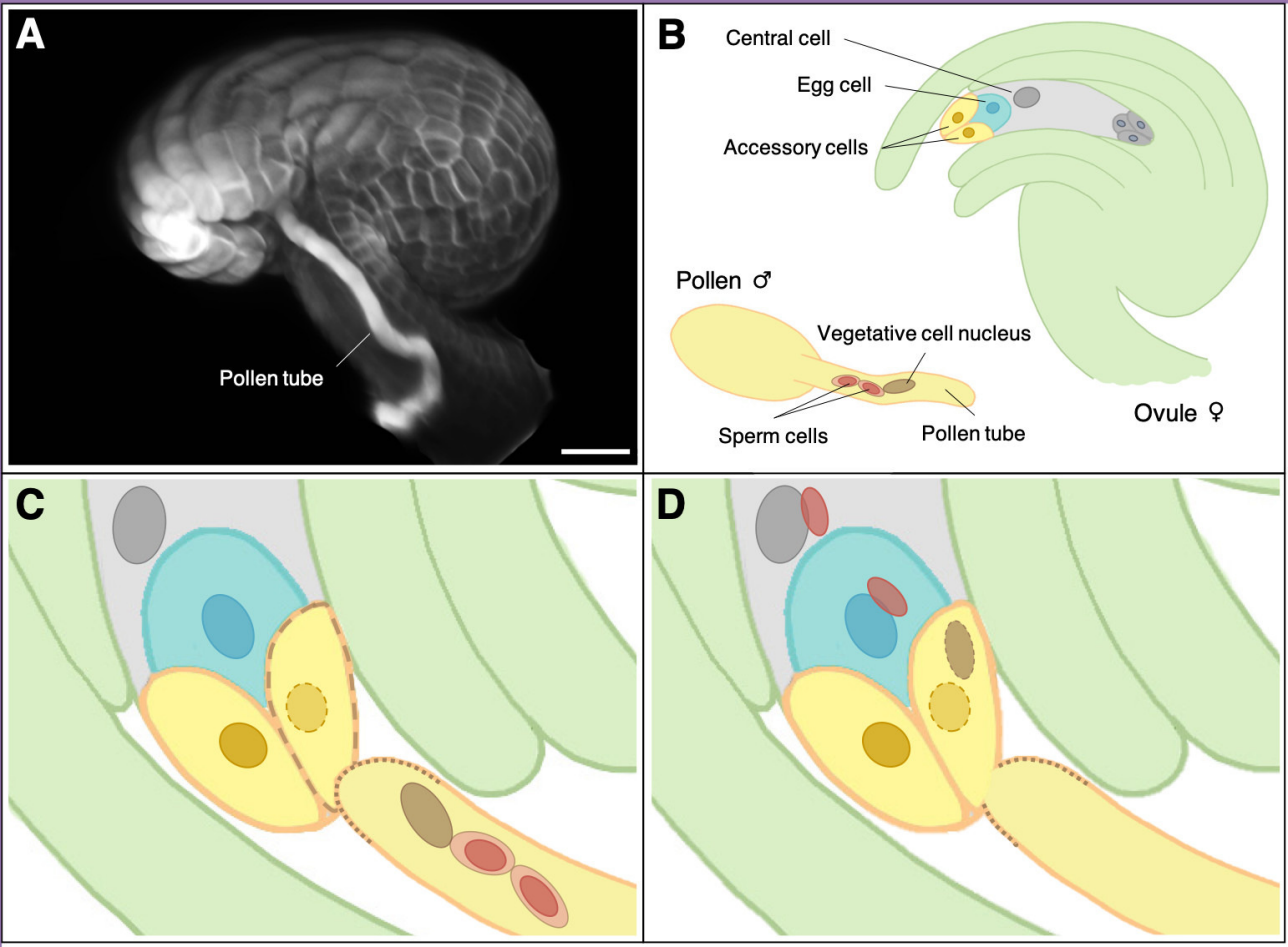
Fertilization in flowering plants. (**A**) Microscopy image of a pollen tube at the opening cleft of a fertilized ovule in the plant species *Arabidopsis thaliana* (scale bar = 20 µm). (**B**) During plant fertilization two sperm cells (red) are carried by a pollen tube (yellow) to the ovule, which contains the egg cell (blue) and the central cell (grey). (**C**) When the pollen tube reaches the opening of the ovule (green) it fuses with one of two accessory cells (yellow), which then starts to degenerate. (**D**) The pollen tube bursts open, releasing the two sperm cells, one of which fertilizes the egg cell to form the zygote, with the other sperm cell fusing with the central cell to form an endosperm cell.

Although polyspermy occurs rarely in egg cells, it happens more frequently in central cells (Grossniklaus, 2017; Köhler et al., 2010; Scott et al., 2008). However, if a central cell receives more than one copy of paternal DNA, this leads to defects in the endosperm that disrupt development and ultimately lead to loss of the seed (Grossniklaus, 2017; Köhler et al., 2010; Scott et al., 2008). The severity of this developmental barrier, also known as the triploid block, depends on the species and often hinders breeding between different species.

In 2017 it was reported that polyspermy of the egg cell in flowering plants results in the development of viable, triploid progeny that contain three sets of chromosomes (Nakel et al., 2017). Now, in eLife, Rita Groß-Hardt and co-workers – including many of the researchers involved in the 2017 work, with Yanbo Mao as first author – report that egg cells undergoing polyspermy can bypass this triploid block (Mao et al., 2020).

The team (who are based at the University of Bremen, Martin Luther University Halle-Wittenberg, and Bielefeld University) used a clever genetic trick to identify viable seeds that contained polyspermy-derived embryos. They found that these seeds essentially originated from a three-parent cross involving two pollen tubes from genetically distinct paternal parents. Further analysis revealed that embryos within the seed were triploid, and contained a maternal genome from the egg cell and a paternal genome from each of the two sperm cells.

Mao et al. also found that the endosperm within these polyspermy-derived seeds had a normal number of chromosomes. This suggests that the egg cell can undergo polyspermy independently from the central cell. Moreover, additional control experiments strongly suggest that selective polyspermy of just the egg cell can bypass the triploid block. In addition, triploid plants produced from the polyspermy-derived seeds could also generate viable polyploid progeny within a single generation.

The evidence presented by Mao et al., together with previous data, strongly suggests that there is no absolute block to egg cell or central cell polyspermy in plants (Nakel et al., 2017; Grossniklaus, 2017; Scott et al., 2008). The selective dual fertilization of just the egg cell described by Mao et al. could explain the fascinating observation that up to 70% of flowering plants are polyploid (Masterson, 1994). Indeed, polyploidy is considered to be of central importance for the evolution and speciation of plants (Van de Peer et al., 2017).

In future studies it will be important to determine the extent of selective egg cell polyspermy among flowering plants, and to establish the ecological and evolutionary relevance of this fascinating process. Bio-engineering of egg cell polyspermy may also facilitate breeding programs and help improve the production of certain crops.
